# Effect of metabolosome encapsulation peptides on enzyme activity, coaggregation, incorporation, and bacterial microcompartment formation

**DOI:** 10.1002/mbo3.1010

**Published:** 2020-02-13

**Authors:** Rokas Juodeikis, Matthew J. Lee, Matthias Mayer, Judith Mantell, Ian R. Brown, Paul Verkade, Derek N. Woolfson, Michael B. Prentice, Stefanie Frank, Martin J. Warren

**Affiliations:** ^1^ Centre for Industrial Biotechnology School of Biosciences University of Kent Canterbury UK; ^2^ School of Biochemistry University of Bristol Bristol UK; ^3^ Wolfson Bioimaging Facility University of Bristol Bristol UK; ^4^ BrisSynBio University of Bristol Bristol UK; ^5^ School of Chemistry University of Bristol Bristol UK; ^6^ Department of Microbiology University College Cork Cork Ireland; ^7^ Department of Biochemical Engineering University College London London UK

**Keywords:** bacterial organelles, cargo, protein aggregation, synthetic biology, targeting

## Abstract

Metabolosomes, catabolic bacterial microcompartments (BMCs), are proteinaceous organelles that are associated with the breakdown of metabolites such as propanediol and ethanolamine. They are composed of an outer multicomponent protein shell that encases a specific metabolic pathway. Protein cargo found within BMCs is directed by the presence of an encapsulation peptide that appears to trigger aggregation before the formation of the outer shell. We investigated the effect of three distinct encapsulation peptides on foreign cargo in a recombinant BMC system. Our data demonstrate that these peptides cause variations in enzyme activity and protein aggregation. We observed that the level of protein aggregation generally correlates with the size of metabolosomes, while in the absence of cargo BMCs self‐assemble into smaller compartments. The results agree with a flexible model for BMC formation based around the ability of the BMC shell to associate with an aggregate formed due to the interaction of encapsulation peptides.

## Graphical abstract

Targeting protein cargo to bacterial microcompartments (BMCs) is reliant on the presence and effectiveness of N‐terminal encapsulation peptides. Three distinct tags were investigated for their ability to target cargo to a recombinant BMC. The three tags resulted in variations in the amount of aggregation and/or colocalization. The level of aggregation was found to influence enzymatic activity, packing, and the size and shape of the structure.

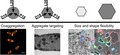

## 1 INTRODUCTION

Bacterial microcompartments (BMCs) are proteinaceous organelles found within a broad range of bacteria (Axen, Erbilgin, & Kerfeld, 2014; Bobik, Lehman, & Yeates, 2015; Kerfeld, Aussignargues, Zarzycki, Cai, & Sutter, 2018). They consist of an outer semipermeable protein shell that surrounds and sequesters a specific metabolic process. BMCs have a diameter of between 100 and 200 nm and can be broadly classified into two main groups based on whether they promote anabolic or catabolic processes. Anabolic BMCs are associated with carbon fixation and house enzymes such as RuBisCO and carbonic anhydrase and are termed carboxysomes (Kerfeld, Heinhorst, & Cannon, 2010; Rae, Long, Badger, & Price, 2013; Yeates, Kerfeld, Heinhorst, Cannon, & Shively, 2008). Catabolic BMCs are associated with the breakdown of metabolites such as propanediol, ethanolamine, and choline, compounds often found in the gut mucosa (Bobik, 2006; Bobik, Havemann, Busch, Williams, & Aldrich, 1999; Cheng, Liu, Crowley, Yeates, & Bobik, 2008; Kerfeld et al., 2018). These degradative BMCs are referred to as metabolosomes (Brinsmade, Paldon, & Escalante‐Semerena, 2005). In all cases studied so far, metabolosomes appear to produce an aldehyde intermediate that is then converted into its alcohol and acid derivatives. The need to encapsulate such metabolic processes appears to protect the cell from potentially toxic intermediates and at the same time promotes metabolic flux (Jakobson, Tullman‐Ercek, Slininger, & Mangan, 2017; Penrod & Roth, 2006; Sampson & Bobik, 2008).

BMCs are composed of several different shell proteins that form either homogeneous hexameric or pentameric tiles, which link together to form the facets and vertices of the macromolecular structure, respectively (Kerfeld et al., 2005; Tanaka et al., 2008). The structure of a small hollow recombinant BMC shell has recently been reported, providing strong evidence on the orientation of the tiles and how they associate to give the overall assembly (Sutter, Greber, Aussignargues, & Kerfeld, 2017). The shell protein tiles have a central pore that is thought to allow metabolites and cofactors into and out of the complex, possibly through a gated mechanism. Many of the metabolic cargo enzymes that are found within the lumen of the BMC are known to contain an N‐terminal, or less frequently a C‐terminal, encapsulation peptide that is around 18–20 amino acids in length, and which forms an amphipathic helix (Aussignargues, Paasch, Gonzalez‐Esquer, Erbilgin, & Kerfeld, 2015; Fan & Bobik, 2011; Fan et al., 2010; Kinney, Salmeen, Cai, & Kerfeld, 2012; Lawrence et al., 2014). For metabolosomes, studies have shown that the N‐terminal 18 amino acids of PduD (D18) (Fan & Bobik, 2011) and PduP (P18) (Fan et al., 2010), as well as the first twenty amino acids of PduL (L20) (Liu, Jorda, Yeates, & Bobik, 2015), can act as targeting peptides that can direct foreign cargo, such as fluorescent proteins, to the BMC.

Research into the biogenesis of BMCs has largely focused on the carboxysomes, which generally form larger and more symmetrical icosahedral structures than the metabolosomes (Iancu et al., 2007; Kerfeld & Melnicki, 2016). Two types of carboxysomes are known, with the β‐carboxysome structural proteins sharing high homology with catabolic BMC proteins. Imaging has shown that the protein shell of β‐carboxysomes can form around an aggregate of protein cargo, with the size and shape of the BMC defined by the architecture of the shell proteins (Cameron, Wilson, Bernstein, & Kerfeld, 2013; Chen, Robinson‐Mosher, Savage, Silver, & Polka, 2013). This core‐first assembly involves the formation of an initial aggregate that gains its shape from the interaction of the shell proteins, which as it closes pinches off the excess aggregate, allowing it to form another BMC. A different assembly process occurs within the α‐carboxysome, where some key proteins help in this fabrication process, including CsoS2 (Cai et al., 2015). This concomitant folding involves the assembly of shell and core proteins together with the chaperoning of core proteins. However, the mechanisms of BMC formation are likely not universal. Metabolosomes are much more irregular in both their size and shape in comparison with carboxysomes. Furthermore, through the recombinant production of BMC shell proteins derived from the propanediol utilization system it has been shown that the shell proteins can self‐assemble into a smaller BMC in the absence of any protein cargo (Mayer et al., 2016; Parsons et al., 2010). Thus, shell proteins can self‐assemble into empty structures.

BMCs represent powerful metabolic units in the form of small bioreactors and as such, they have caught the attention of metabolic engineers who see these organelles as a way to redesign specific aspects of cellular metabolism for the production of fine and commodity chemicals (Frank, Lawrence, Prentice, & Warren, 2013; Kim & Tullman‐Ercek, 2013; Lawrence et al., 2014; Lee, Palmer, & Warren, 2018; Liang, Frank, Lunsdorf, Warren, & Prentice, 2017; Mayer et al., 2016). Key to the redesign of BMCs with alternative metabolic functions is the ability to encapsulate new pathways into the structures. Evidence that BMCs can be reengineered has come from a proof‐of‐principle study whereby a pyruvate decarboxylase (PDC) and an alcohol dehydrogenase were directed to an empty BMC through the addition of encapsulation peptides (Lawrence et al., 2014). The resulting BMC was found to have the ability to convert pyruvate into ethanol.

Although a broad range of potential encapsulation peptides has been identified, comparative studies on their ability to direct cargo to BMCs have been limited. Screens to report improved changes to the encapsulation peptide have been reported, but these are based on the use of fluorescent proteins and their localization to puncta in cells by fluorescent microscopy (Kim & Tullman‐Ercek, 2014). However, as encapsulation peptides promote aggregation, which can be visualized also as fluorescent foci, the use of fluorescent puncta as a means to judge localization does not differentiate between protein aggregation and BMC encapsulation (Lee, Palmer, et al., 2018).

Herein, we describe research that was undertaken to develop improved methods to quantitate the incorporation of cargo into BMCs. Initially, the ability of the *Citrobacter freundii* PduD, PduL, and PduP to direct cargo to an empty recombinant *C. freundii* Pdu BMC was investigated. Thereafter, the *C. freundii* D18, L20, and P18 tags were investigated for their effect on the activity of a range of PDCs to inform on the choice of the tag. The peptides were also studied for their ability to coaggregate different proteins in vivo. We demonstrate that metallothionein can be used to follow aggregation and encapsulation as it is easily identifiable by TEM due to its increased electron density, which is most likely caused by its metal binding capacity. Finally, we present electron tomography studies of empty recombinant *C. freundii* Pdu BMCs and metallothionein targeted to these BMCs. Collectively, this work demonstrates a correlation between cargo protein aggregation, the level of assimilation into BMCs, and their resulting size.

## 2 RESULTS

### 2.1 Targeting of native enzymes into recombinant Pdu BMCs

**Figure 1:**
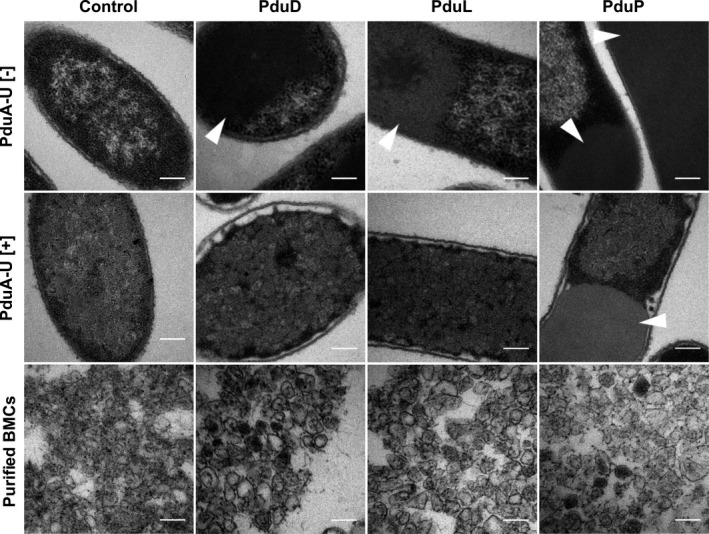
Encapsulation of native proteins in recombinant Pdu BMCs. Electron micrographs of *Escherichia coli* cells producing PduD, PduL, or PduP in the absence (top panel) and presence (middle panel) of a minimal BMC shell system (PduA‐U). The BMCs extracted from strains in the middle panel are also shown. The control sample contains an empty vector. Arrows indicate areas of protein aggregation. All scale bars are 200 nm

**Figure A1:**
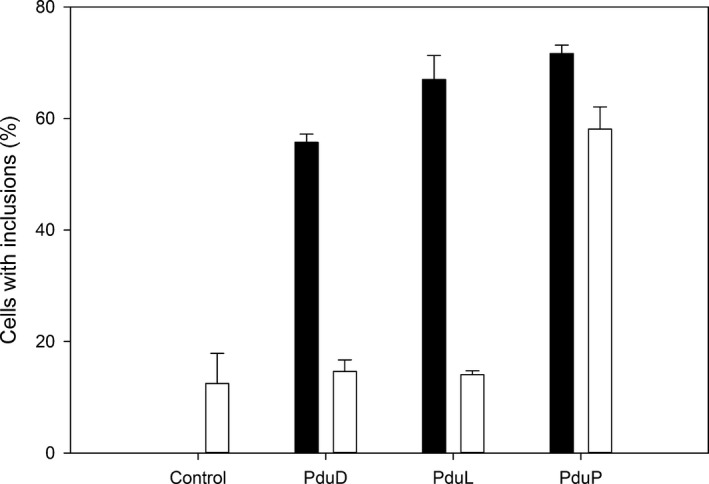
Percentage of cells expressing the relevant proteins or an empty vector (control) containing intracellular aggregates. Black bars indicate no compartment control, white bars—coproduced with PduA‐U. Error bars equal one standard deviation between three separate counts of a single biological repeat (*n* ≥ 330)

To understand the relative abilities of the encapsulation peptides found on *C. freundii* PduD (Fan & Bobik, 2011), PduL (Liu et al., 2015), and PduP (Fan et al., 2010) to mediate cargo encapsulation into a recombinant *C. freundii* Pdu BMC, we first sought to look at how well these proteins were integrated into an empty BMC. We therefore individually produced PduD, PduL, and PduP in the presence and absence of empty Pdu BMCs (PduA‐U) (Parsons et al., 2010). When these BMC‐associated enzymes were produced in the absence of compartments, large inclusion bodies were observed in a high proportion of cells (PduD: 55.8%; PduL: 67.0%; PduP 71.7%; Figure A1 (Appendix 1)) with no such structures present in an empty vector control strain (Figure 1). This observation is consistent with the prediction that these proteins self‐aggregate.

**Figure A2:**
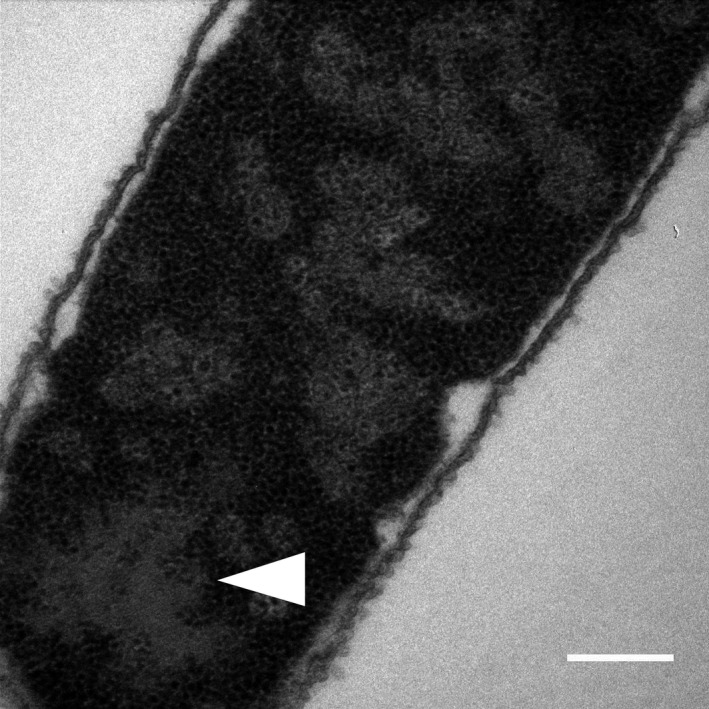
Electron micrograph of PduD coexpressed with recombinant BMCs (PduA‐U). Arrow showing aggregation which is phenotypic for misfolded BMCs. Scale bar is 0.2 µm

**Figure A3:**
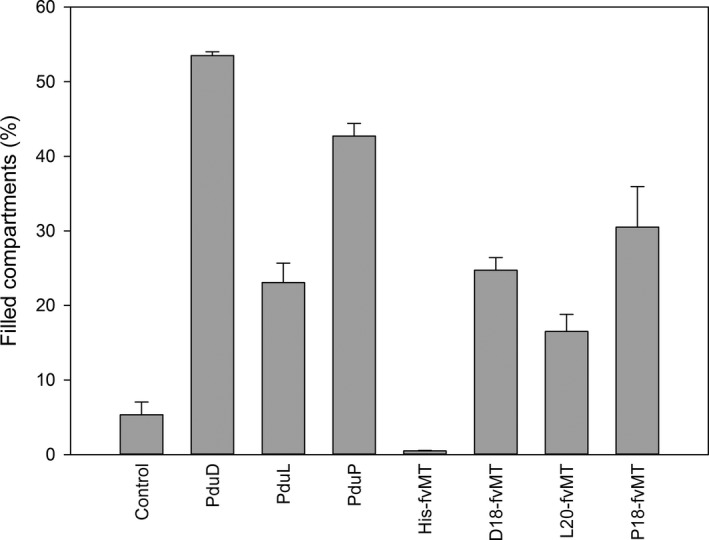
Percentage of purified intact BMCs coproduced with the relevant proteins showing electron density within the lumen of the compartment indicative of successful targeting. Error bars equal one standard deviation between three separate counts of a single biological repeat (*n* ≥ 440)

Coproduction of PduD or PduL with PduA‐U resulted in a decrease in the observed aggregation (Figure 1 and Figure A1 (Appendix 1)). The inclusions observed in these cells were phenotypic of misfolded BMC shell proteins (Figure A2 (Appendix 1)), suggesting that the majority of PduD and PduL were encapsulated. However, coproduction of PduP with BMCs only showed a slight decrease in the percentage of cells containing inclusion bodies (Figure A1 (Appendix 1)), although a high proportion of cells (58.1%) still contained inclusions, suggesting lower encapsulation efficiency or greater aggregation rate. Electron microscopy analysis was also carried out on purified BMCs from these strains (Figure 1). Areas of darker staining within the isolated BMCs indicate that the BMCs contain protein cargo and thus the number of BMCs containing darker staining can be used to gauge targeting to the lumen of intact microcompartments (Figure A3 (Appendix 1)). The darker electron density within the BMC is therefore indicative of protein encapsulation. These BMCs were compared to BMCs produced in the absence of cargo. Electron‐dense regions were observed in the lumen of BMCs isolated from the strains producing PduD, PduL, or PduP, suggesting that all three proteins are incorporated into the recombinant BMCs. The efficiency of protein incorporation displayed a degree of variation with both PduP and PduD showing a higher level of assimilation in comparison with PduL.

### 2.2 Effect of Pdu BMC encapsulation tags on three different pyruvate decarboxylases

**Figure A4:**
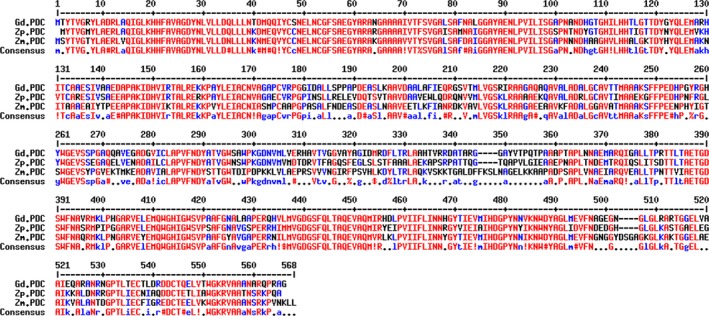
Protein sequence alignment of the analyzed pyruvate decarboxylases from *Gluconacetobacter diazotrophicus* (Gd.PDC), *Zymobacter palmae* (Zp.PDC), and *Zymomonas mobilis* (Zm.PDC). Generated using the MultAlin tool (Corpet, 1988)

Previously, we reported the effect of encapsulation tags on the activity of pathway enzymes associated with propanediol synthesis (Lee, Brown, Juodeikis, Frank, & Warren, 2016). Herein, we investigated the effect of different encapsulation peptides on the activity of PDC, an enzyme we had previously shown could be targeted to a recombinant BMC (Lawrence et al., 2014), and explored whether the effects were identical on three homologous PDCs. The selected PDCs have a high level of protein sequence similarity (Figure A4 (Appendix 1)) as this would allow us to determine whether the addition of encapsulation peptides has a reproducible effect on enzymatic activity.

**Figure 2:**
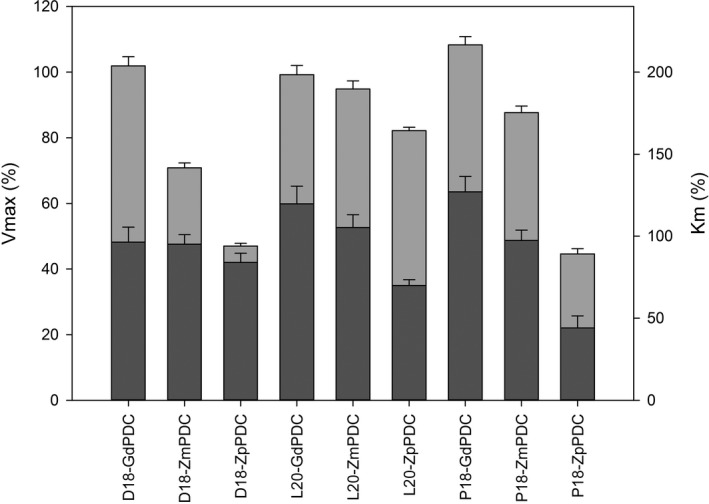
Comparison of the kinetic values of modified PDCs. Encapsulation peptides (D18, L20, and P18) were fused to three distinct PDCs. After recombinant production and purification, the kinetic parameters of the various encapsulation‐fused PDCs were measured in terms of V_max_ (left scale bar) and K_m_ (right scale bar) and expressed as a percentage of the activity of the PDC without the encapsulation tag. Light gray bar—*V_max_*; dark gray bar—*K_M_*. Assays were carried out in triplicate; error bars equal one standard deviation

To undertake this comparison, we looked at the effect on enzyme activity after fusing three different BMC encapsulation peptides (D18, L20 and P18) onto PDCs from *Gluconacetobacter diazotrophicus* (GdPDC), *Zymomonas mobilis* (ZmPDC), and *Zymobacter palmae* (ZpPDC). The encapsulation peptides were fused to the N‐terminus of the PDCs together with a hexahistidine linker and a thrombin cleavage site. The control construct contained only a PDC with a hexahistidine linker and a thrombin cleavage site. The activities of the purified tagged (D18, L20, P18) and untagged enzymes were followed in a linked assay. A comparison of the activities is shown in Figure 2.

The data show that despite a high level of sequence similarity between the enzymes (Figure A4 (Appendix 1)), particularly at the N‐terminus where the encapsulation peptide is located, the different tags have a variable effect on activity. For instance, in comparison with the His‐only control, the different tags did not greatly affect GdPDC activity, whereas the *V_max_* of ZpPDC was reduced by around 50% when the protein was tagged with a D18 or P18 encapsulation peptide. Overall, the L20 tag was found to have the least disruptive effect on the activity of the enzymes. The activity results suggest that the effect of the encapsulation tags is not dependent on sequence similarity, making it difficult to predict the behavior of a tagged enzyme.

### 2.3 A comparative analysis of encapsulation efficiencies

Although mutagenesis approaches to improving the ability of encapsulation peptides to promote assimilation have been reported (Kim & Tullman‐Ercek, 2014), no direct comparison has been undertaken to determine the relative targeting efficiencies of the various encapsulation peptides that have been used to direct non‐natural cargo to recombinant BMCs. However, knowledge on the relative effectiveness of the different peptides would be a key facilitator of BMC technology in an industrial setting.

**Figure 3:**
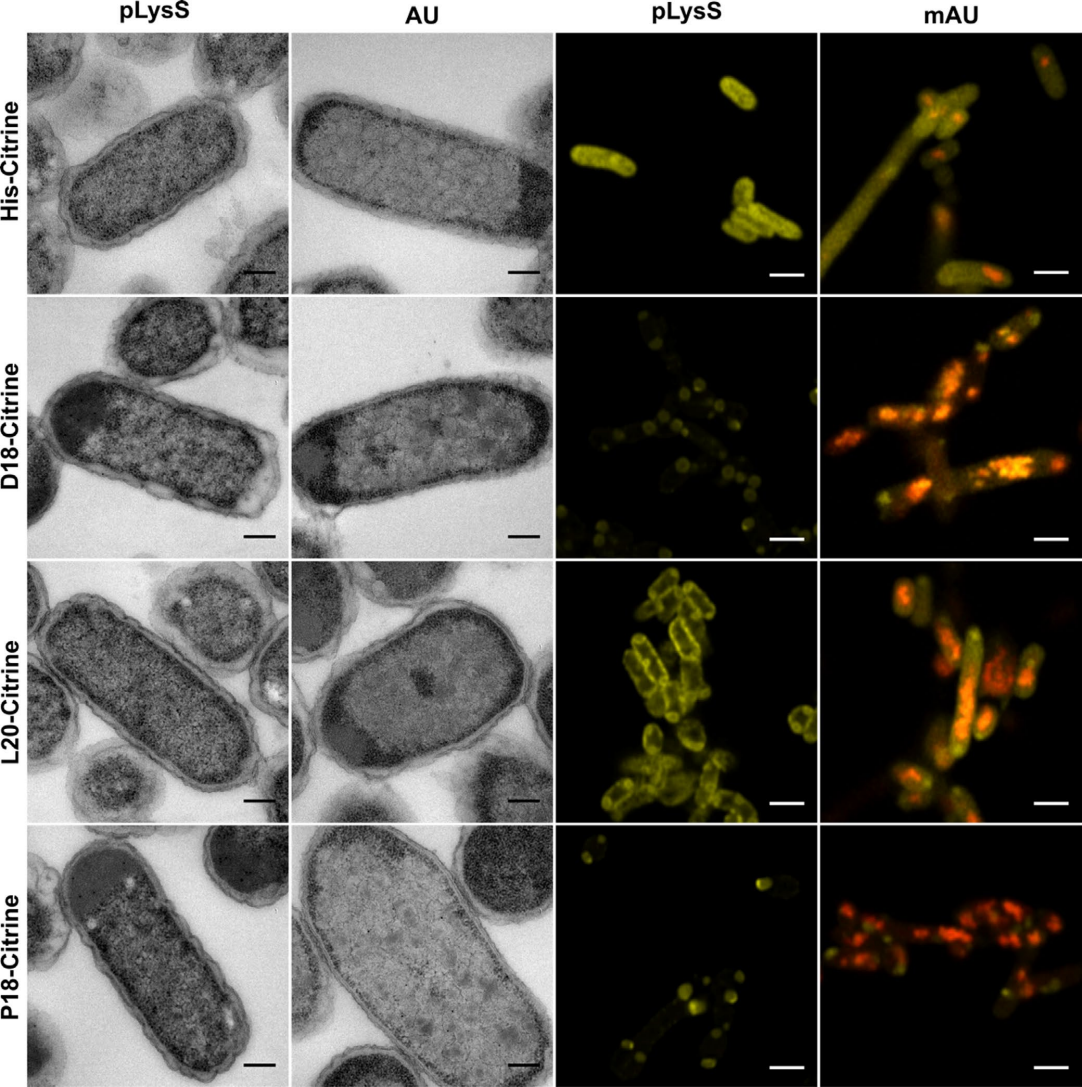
Targeting of fluorescent proteins to recombinant Pdu BMCs. Transmission electron micrographs (left two columns) and confocal microscopy (right two columns) images of cells expressing differentially tagged Citrine in the presence and absence of mCherry‐tagged BMCs (mA‐U). Cells containing the empty pLysS vector are unable to produce BMCs, while those containing mA‐U within the pLysS vector produce BMCs with an mCherry tag as evidenced by red puncta within the cytoplasm. The production of Citrine is visualized by the presence of yellow. Superimposition of red and yellow is indicative of colocalization. Scale bars in TEM micrographs show 0.2 µm and in confocal images 2 µm

To investigate the encapsulation efficiencies of the D18, L20, and P18 encapsulation peptides, we utilized a combination of confocal fluorescence microscopy and immuno‐TEM. A yellow fluorescent protein (Citrine) was fused to the D18, L20, and P18 tags. The genetic constructs for these fusion proteins were coexpressed with either recombinant BMCs containing an mCherry‐fused PduA (mA‐U) (Parsons et al., 2010) or an empty vector control (pLysS) and analyzed (Figure 3).

Expression of either D18‐ or P18‐tagged Citrine, in the absence of BMCs, resulted in the appearance of inclusion bodies, which were visible by both microscopy methods. In contrast, the L20‐tagged protein appeared more soluble, but was observed to localize to the periphery of the cell. Analysis of a strain producing only mA‐U revealed the presence of red puncta within the cytoplasm, an observation that has been attributed to the formation of BMCs within the cell (Parsons et al., 2010). When coproduced with mA‐U, both D18‐ and P18‐tagged Citrine localized to these red punctate regions, which is indicative of colocalization with BMCs.

**Figure A5:**
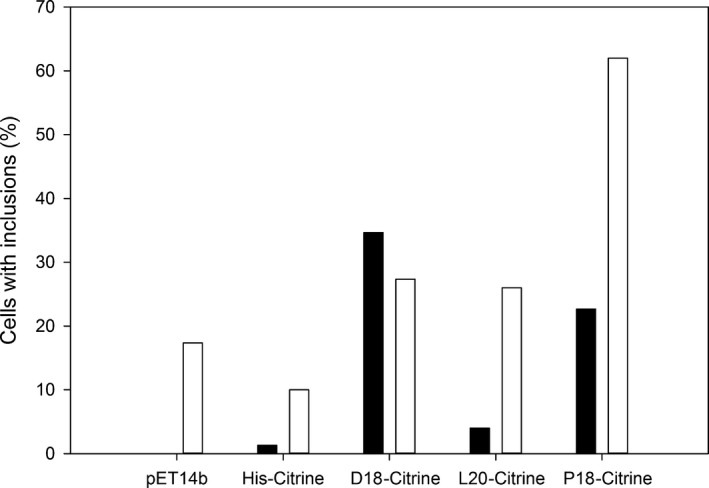
Quantification of aggregates observed in cells producing Citrine targeted to BMCs (*n* = 150). Black bars indicate no compartment control, while white bars indicate coproduction with mA‐U. Only one count was carried out

In contrast, the L20‐tagged protein did not appear to target specifically to the punctate regions of the cells, although there was a change in the observed phenotype with the signal more evenly dispersed throughout the cell. Quantification of the inclusion bodies present in these strains by TEM (Figure A5 (Appendix 1)) did not show a reduction when coproduced with BMCs as has been observed previously with the full‐length PduD, PduL, and PduP proteins (Figure A1 (Appendix 1)). The difference may be explained by the modification of PduA in the mA‐U construct as the addition of the mCherry label to PduA may alter BMC stability or result in increased shell protein aggregation.

### 2.4 Coaggregation of tagged proteins

**Figure 4:**
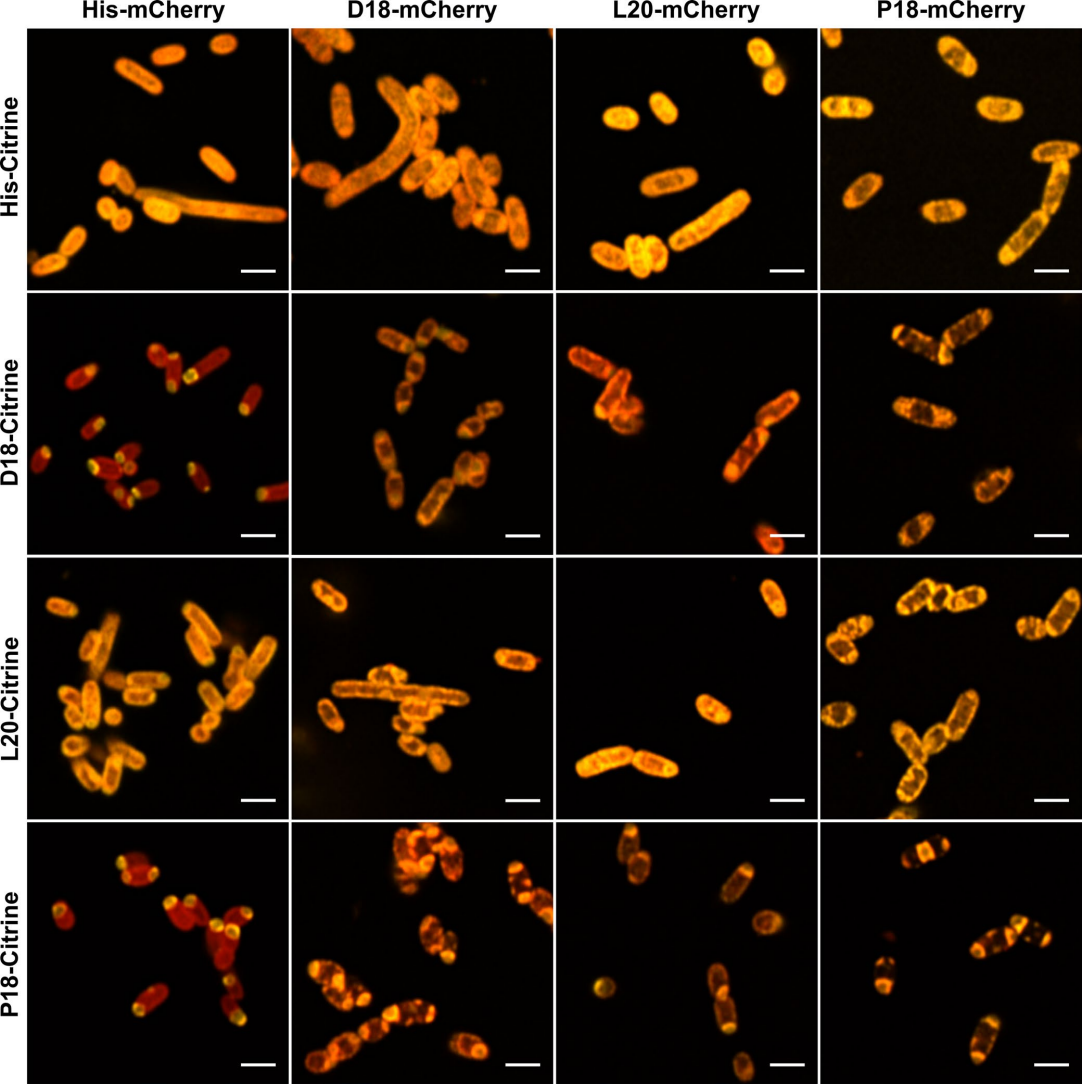
Colocalization of fluorescent proteins tagged with encapsulation peptides. Confocal microscopy was performed on encapsulation peptide‐fused fluorescent proteins to determine whether the encapsulation peptides interact with themselves or each other to coaggregate. Citrine (yellow) and mCherry (red) fluorescent proteins tagged with or without the various encapsulation peptides (D18, L20, or P18) were coproduced and imaged as shown. All scale bars are 2 µm

It would appear that the presence of D18 and P18 on proteins aids in the formation of cellular inclusion bodies. Previous work had also shown that the expression of 4 proteins involved in a 1,2‐propanediol synthesis pathway tagged with encapsulation peptides results in the formation of a single large inclusion body, which is thought to contain all of the tagged components (Lee et al., 2016). To investigate this aggregation phenotype more thoroughly, we coproduced two fluorescent proteins (mCherry and Citrine) containing the various Pdu targeting tags to see whether the fluorescent signals localize to the same region of the cell and form puncta indicative of protein aggregation (Figure 4).

**Figure A6:**
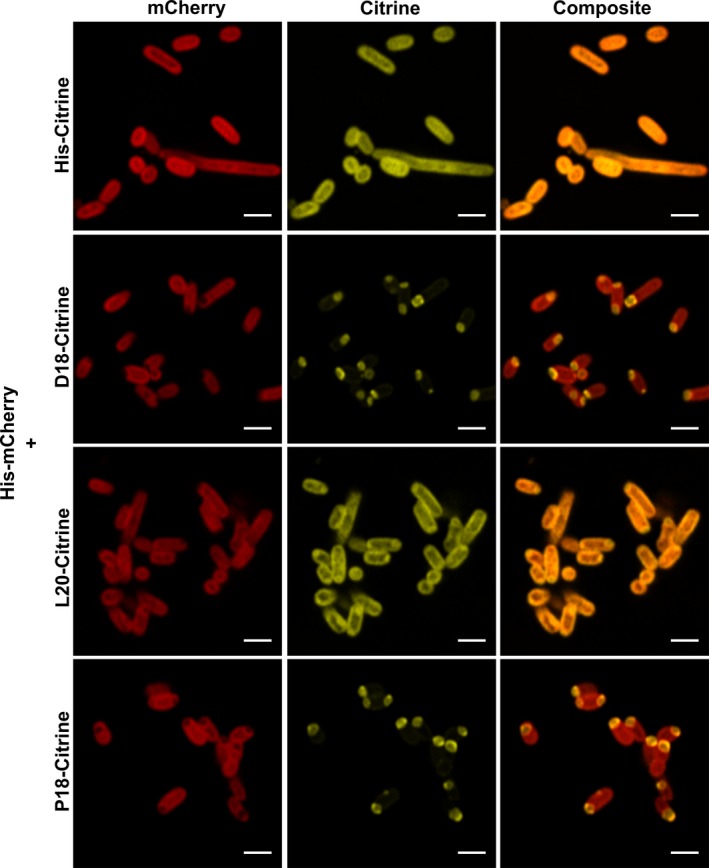
Coexpression of His‐tagged mCherry with Citrine tagged with different BMC targeting peptides. Scale bars are 2 µm

**Figure A7:**
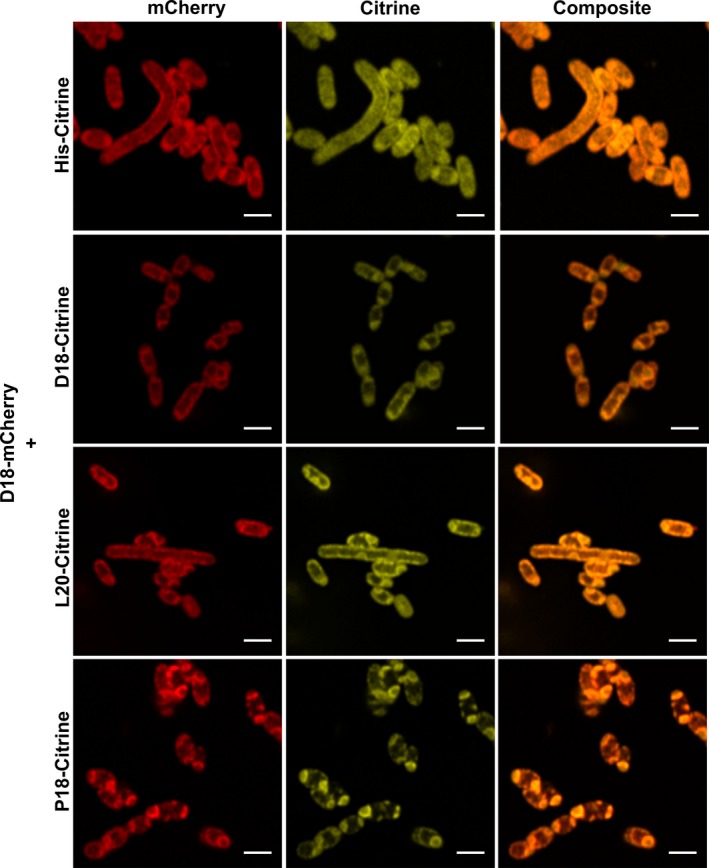
Coexpression of D18‐tagged mCherry with Citrine tagged with different BMC targeting peptides. Scale bars are 2 µm

**Figure A8:**
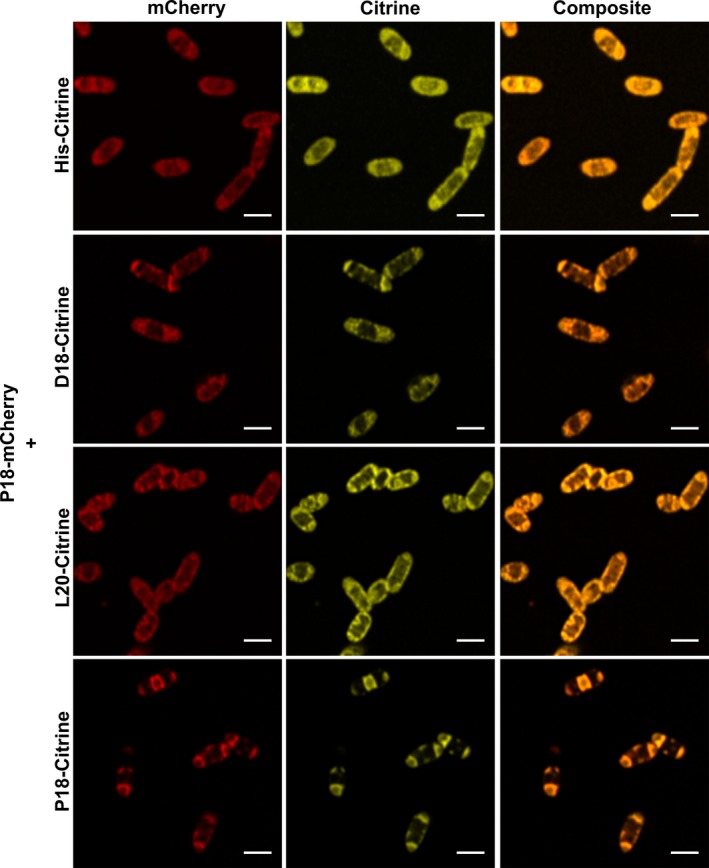
Coexpression of P18‐tagged mCherry with Citrine tagged with different BMC targeting peptides. Scale bars are 2 µm

**Figure A9:**
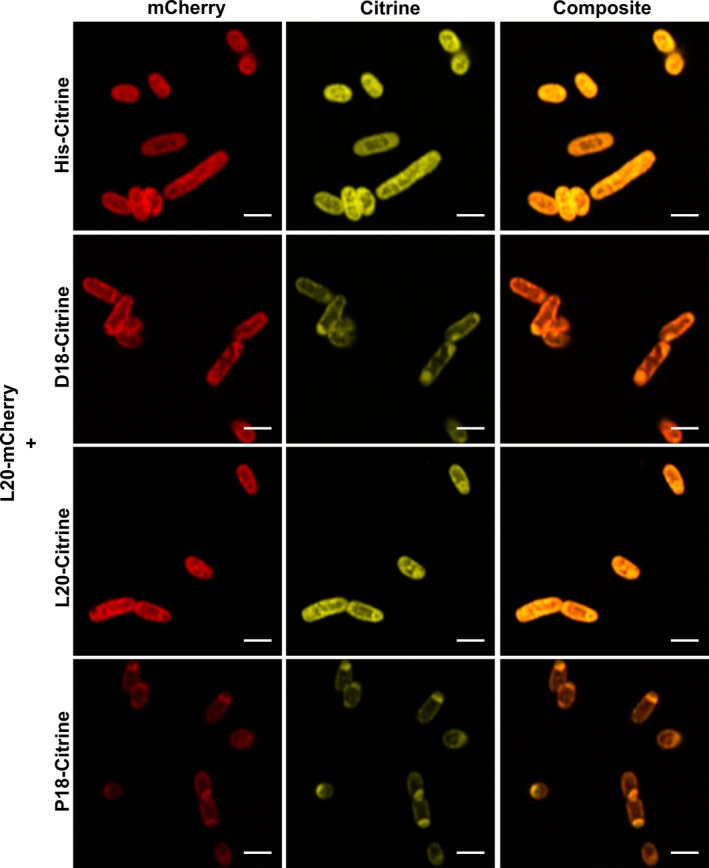
Coexpression of L20‐tagged mCherry with Citrine tagged with different BMC targeting peptides. Scale bars are 2 µm

Coexpression of His‐mCherry with His‐Citrine resulted in dispersed fluorescence throughout the cytoplasm (Figure A6 (Appendix 1)). However, the introduction of either a D18 or P18 tag onto the N‐terminus of mCherry resulted in its aggregation (Figures A7 and A8 (Appendix 1)), while the His‐Citrine fluorescent signal remained cytoplasmic. In contrast, the production of L20‐mCherry resulted in mostly cytoplasmic fluorescence suggesting that this targeting peptide has a lower predisposition for aggregation (Figure A9 (Appendix 1)).

When the fluorescent proteins were tagged with either the D18 or P18 encapsulation peptides, coaggregation was observed, demonstrating that the tagged components are aggregated into a single inclusion body rather than separate bodies (Figure 4). Again, the L20‐tagged fluorescent proteins appeared much more soluble than their P18 or D18 equivalents; although when the L20‐fluorescent proteins were coproduced together with a different fluorescent protein tagged with either a D18 or P18 tag, the fluorescent signals appeared to colocalize with the punctate aggregates (Figure 4 and Figure A9 (Appendix 1)). Thus, it would appear that while the L20 encapsulation peptide is more soluble, it interacts with D18 and P18 tags leading to coaggregation.

### 2.5 Targeting metallothionein to recombinant Pdu BMCs

**Figure 5:**
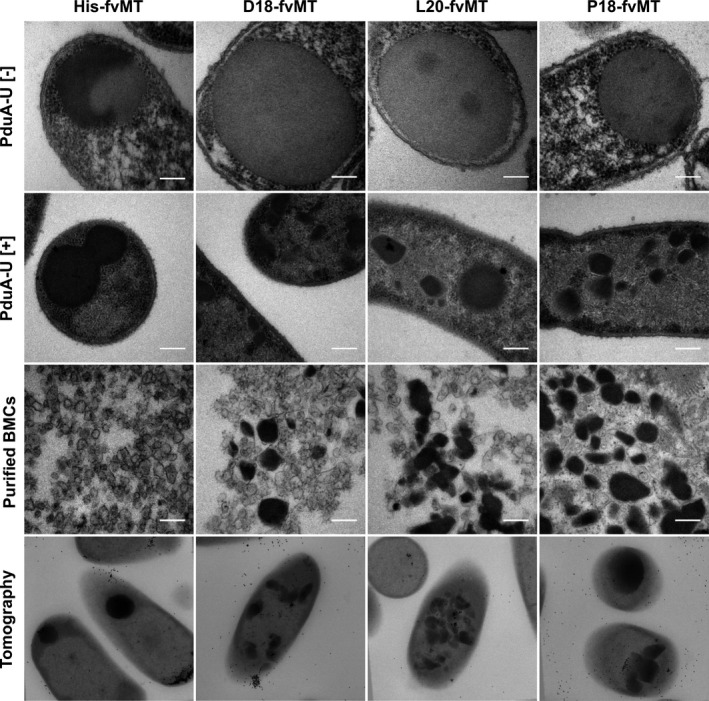
Production of metallothionein fused to various encapsulation peptides in the presence and absence of BMCs. TEM analysis of cells producing metallothionein tagged with the D18, L20, and P18 encapsulation peptides in the absence (top row) and presence (second row) of PduA‐U BMCs. TEM of isolated BMCs from the strains in row 2 is shown in row 3. The bottom row shows TEM tomography of sections cells from row 2—see Video S1 at https://doi.org/10.5281/zenodo.3611413 for full tomography data. All scale bars are 200 nm

To study further the efficiency of targeting recombinant proteins into BMCs, we utilized a metal binding protein, metallothionein, due to its propensity to bind a variety of metal ions (Kagi & Schaffer, 1988) making it comparatively easy to visualize within the cell by TEM when aggregated. The idea behind the metallothionein approach is that it should allow the opportunity to observe encapsulated aggregates in whole cell sections as opposed to relying on in vitro data obtained from purified compartments, allowing a comparison of their relative size, shape, and volume. We therefore tagged metallothionein from *Fucus vesiculosus* (Morris, Nicolaus, Sampson, Harwood, & Kille, 1999) (fvMT) with the three different encapsulation peptides as well as a His‐tag control. These constructs were coproduced with PduA‐U, and the resulting strains, as well as purified BMCs, were analyzed by TEM (Figure 5). As expected, the use of fvMT did indeed allow the protein aggregates to be easily identified by TEM due to the increased electron density. Thin sections of cells from strains producing only the His‐fvMT, D18‐fvMT, L20‐fvMT, and P18‐fvMT all showed the presence of a large aggregate in the absence of BMCs. When this was repeated with strains producing not only the tagged fvMT but also empty compartments (PduA‐U), smaller bodies with sharp edges were observed, which is indicative of encapsulation into BMCs.

**Figure A10:**
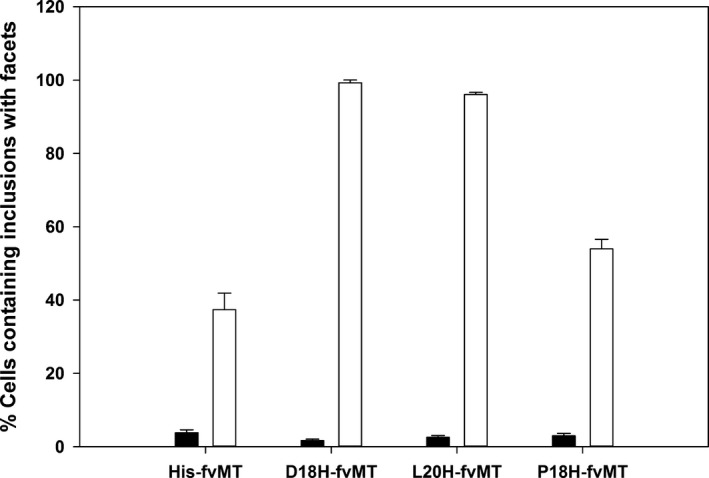
Quantification of aggregates containing facets observed in cells producing fvMT targeted to BMCs. Black bars indicate no compartment control, white bars—coproduced with PduA‐U. Error bars equal one standard deviation between three separate counts of a single biological repeat (*n* ≥ 220)

**Figure A11:**
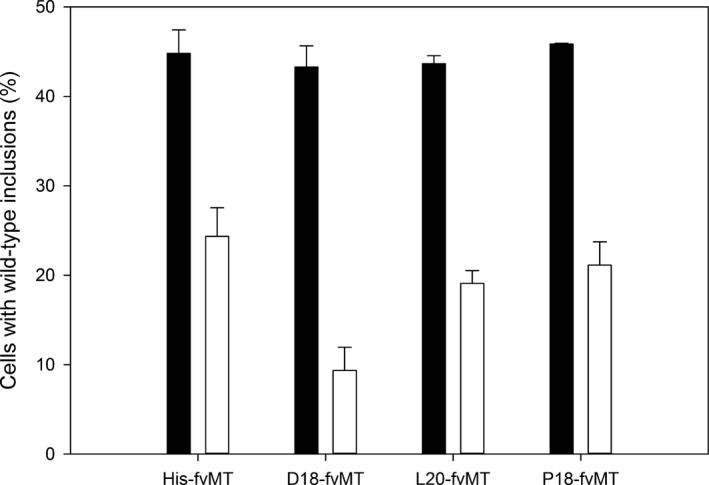
Quantification of overall aggregation observed in cells producing fvMT targeted to BMCs. Black bars indicate no compartment control, white bars—coproduced with PduA‐U. Error bars equal one standard deviation between three separate counts of a single biological repeat (*n* ≥ 435)

**Figure A12:**
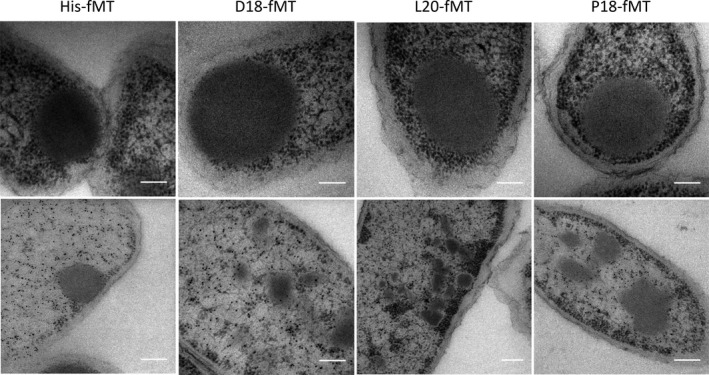
Immuno‐TEM of targeted fMT constructs produced with or without compartments showing the localization of PduA. First row—cells with no PduA‐U; second row—cells with protein coproduced with PduA‐U. All scale bars are 200 nm

Quantification indicated that the presence of either the D18 or L20 peptides on the fvMT resulted in efficient encapsulation (99.3% and 96.1%, respectively; Figure A10 (Appendix 1)), in that the vast majority fvMT was found associated with BMCs rather than an inclusion body. By way of contrast, the P18‐tagged fvMT shows only roughly half that level (54.0%) with an even distribution between aggregates and BMCs. The presence of an encapsulation peptide resulted in an overall decrease in the presence of nonencapsulated aggregation with the D18 tag proving to be the most effective (Figure A11 (Appendix 1)). We also observed a high proportion of empty BMCs present in the cells (Figure 5, third row), suggesting that the ratio of cargo to shell production in a recombinant system requires further optimization. Significantly, the BMCs observed with the tagged fvMT all appear much larger than the BMCs isolated from the untagged fvMT. To demonstrate that the angular electron‐dense bodies are BMCs, the thin sections of the various strains were analyzed by immuno‐TEM using an anti‐PduA antibody (Figure A12 (Appendix 1)). If the angular structures are BMCs, then we would expect them to cross‐react with the anti‐PduA antibody, which was observed and is consistent with these aggregates being surrounded by a BMC shell.

**Figure A13:**
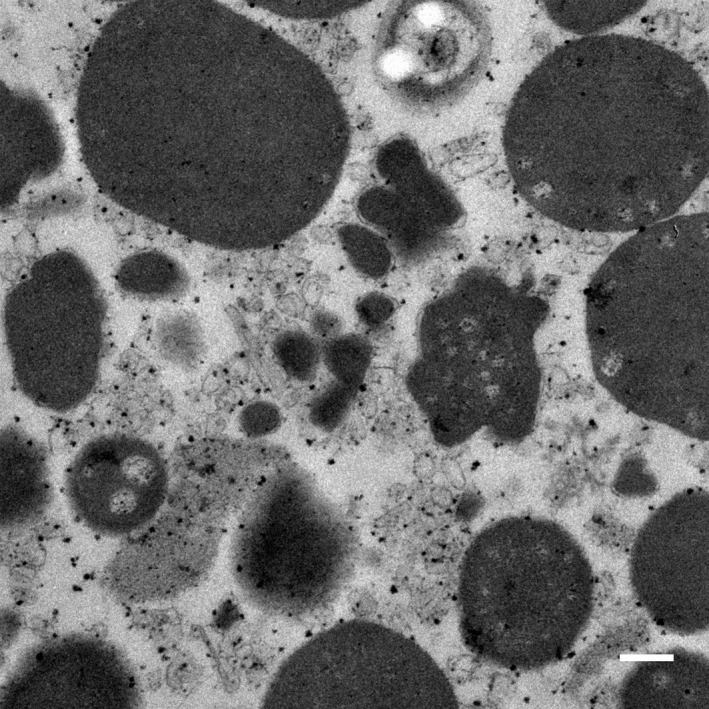
Large aggregates observed in microcompartment purification. Scale bar is 0.2 µm

The purified compartments produced in these various strains were also analyzed by TEM (Figure 5 and Figure A12 (Appendix 1)). Significantly, the BMCs coproduced with His‐fvMT did not appear to have any cargo present after purification. The BMCs isolated from strains with the fvMT tagged with the encapsulation peptides had various observed incorporation efficiencies (D18 – 24.7%; L20 – 16.5%; P18 – 30.5%). The purified BMCs coproduced with P18‐fvMT also contained what appeared to be large inclusion bodies (Figure A13 (Appendix 1)). We suggest that these are large P18‐fvMT inclusions that copurify nonspecifically with the compartments. Inclusion bodies like these are likely to pull down during BMC purification due to their large size, highlighting the importance of using TEM for this analysis in addition to standard Western blot analysis to define encapsulation of proteins into BMCs.

**Figure 6:**
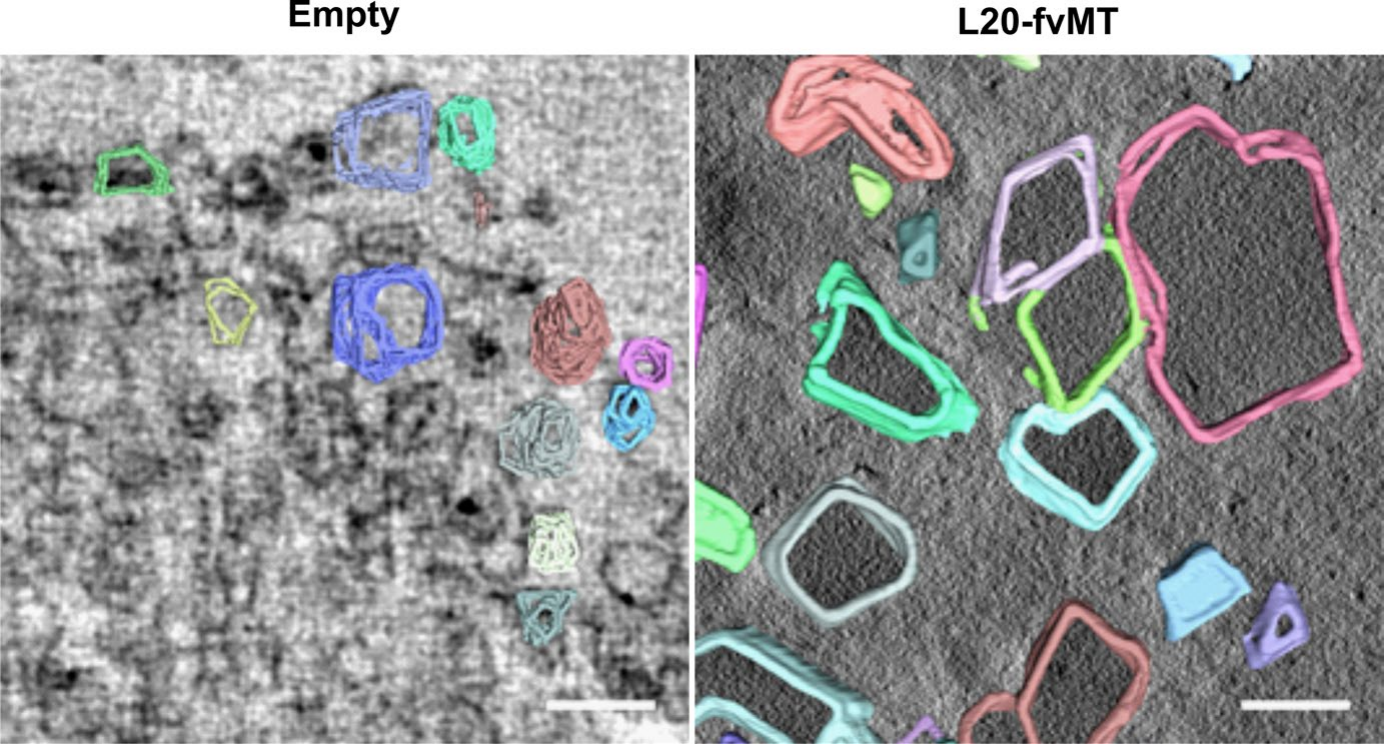
3D reconstructions of Pdu microcompartments. Traced tomograms of PduA‐U (left) and L20H‐fMT PduA‐U (right). All scale bars are 200 nm

The fvMT‐producing strains were further analyzed by electron tomography to gain better visualization of the three‐dimensional topography of these structures (Figure 5 and Video S1 at https://doi.org/10.5281/zenodo.3611413). This approach revealed the structures formed by fvMT and BMC shell proteins are remarkably varied in size, shape, and volume. Furthermore, AMIRA software was used to gather quantitative data analyzing enclosed structures in these tomograms (Weber et al., 2012). This approach allowed us to gain further insight into the 3D structure (Figure 6) of empty recombinant BMCs (Video S2 at https://doi.org/10.5281/zenodo.3611413) and recombinant BMCs containing L20‐fvMT (Video S3 at https://doi.org/10.5281/zenodo.3611413) allowing us to quantitate the volume (empty: 54,900 ± 11,013 nm^3^ (*n* = 29); L20‐fvMT: 336,411 ± 177,722 nm^3^ (*n* = 60)) and the largest diameter (empty: 61.77 ± 15.38 nm (*n* = 29); L20‐fvMT: 127.51 ± 60.97 nm (*n* = 60)) of these structures. These data suggest an average volumetric expansion of around 6 times to accommodate L20 tagged fvMT. Modeling suggests that strong cargo–cargo interactions lead to an increase in compartment size, while strong shell–shell interactions lead to smaller, better defined structures (Mohajerani & Hagan, 2018; Perlmutter, Mohajerani, & Hagan, 2016). Our observations therefore are indicative of an assembly mechanism dominated by strong cargo–cargo interactions. These findings could also explain the previously observed variability in BMC size and shape (Mayer et al., 2016).

## 3 DISCUSSION

It had previously been observed that the removal of the N‐terminal extension found on some of the diol dehydratases linked with BMC‐catabolic processes improved their solubility, allowing for their structure determination (Fan et al., 2010). Studies on the structure of the P18 peptide revealed that it forms an amphipathic helix that encourages self‐association through a coiled‐coil interaction (Lawrence et al., 2014). In this way, the encapsulation peptides promote protein aggregation. The protein aggregate must then be able to interact with the luminal side of the BMC shell. Indeed, the identification of P18 as an encapsulation peptide also led to suggestions that the peptide may interact with one particular component of the shell, PduA. Specifically, modeling studies suggested that the P18 peptide could interact with a helical region of PduA (Fan, Cheng, Sinha, & Bobik, 2012). However, this region of PduA is on the concave side of the PduA hexamer. If the recent structure determination of a recombinant BMC from *Haliangium ochraceum* (Sutter et al., 2017) is an accurate representation of a wild‐type compartment, and all the shell proteins have their concave side facing into the cytoplasm, then this encapsulation model will need to be modified to explain how the cargo protein becomes localized within the lumen.

By studying the production of PduD, PduL, and PduP in the presence and absence of BMCs, we have shown that, individually, PduD, PduL, and PduP all form aggregates in the cell. However, in the presence of BMCs the majority of PduD and PduL become incorporated into the BMCs, as viewed by higher electron density within isolated BMCs and the reduction in the observable intracellular aggregation. High levels of intracellular aggregation were still observed for PduP, although targeting was confirmed within BMCs. Using TEM for investigation of higher density within the BMCs represents an important technique to help in the validation that protein cargo is being localized within the BMC. This can be used to help support the evaluation of localization with fluorescent proteins and the presence of fluorescence puncta within cells.

It was interesting to observe the effect of the addition of encapsulation peptides to a range of PDCs from different organisms. We thought that there may be some common effects of these encapsulation peptides on the activities of the homologous enzymes. For instance, we would have predicted that L20 would have less of an effect on activity than the other tags. We could not observe any specific trend with the peptide. The tags had little to no effect on the activity of the PDC from *G. diazotrophicus*, whereas the P18 and D18 tags had a clear effect on the *Z. palmae* PDC. This means that predicting the effect of the addition of a targeting peptide to an enzyme is likely to be very challenging.

Attachment of the encapsulation peptides to fluorescent proteins allowed for a study of their colocalization in the absence of BMCs. The work shows that fluorescent proteins containing targeting tags can coaggregate together prior to encapsulation. These results suggest a possible assembly mechanism where cargo proteins coaggregate together prior to encapsulation.

To gauge the effect of targeting to BMCs, one really needs to be able to see whether protein has been internalized within the structure and also to be able to measure the size and shape of the compartment. We were able to achieve this through the use of metallothionein where aggregated protein is easily identifiable within the cell due to its extra density caused by the acquisition of metal ions. Using fvMT, we were able to demonstrate that all the encapsulation tags, when attached to the protein, cause encapsulation within recombinant BMCs. These studies are all consistent with a model for BMC formation whereby shell proteins interact with an initial aggregate. If the aggregate forms too quickly, then the BMC cannot keep pace with the aggregate and the BMC does not encapsulate the cargo which results in the formation of a large inclusion within the cell as seen with P18‐fvMT (Figure 5 and Figure A10 (Appendix 1)). However, if enough shell protein is available, the shell is able to form around the aggregate with high flexibility. The extent of fvMT aggregation was differentially influenced by the various tags. L20‐encapsulated fvMT aggregates were smallest, followed by D18, while P18 fvMT produced the largest structures, which was often observed to form unencapsulated inclusions. The extent of aggregation caused by the tags seems to be conserved for all experiments, suggesting that P18 tag causes the largest amount of aggregation, followed by the D18 tag, while L20 tag does not cause large amounts of aggregation, but can still effectively target to compartments whether the protein is able to aggregate by itself, as apparent in the fvMT experiments.

In the absence of any cargo, the shell proteins are able to associate together and generate comparatively small structures. Overall, the size and shape of BMCs appear to be primarily dictated by cargo–cargo and shell–cargo interactions, explaining why catabolic BMCs have such a varied topology as predicted by computational and theoretical modeling (Mohajerani & Hagan, 2018).

## 4 EXPERIMENTAL PROCEDURES

### 4.1 Molecular biology and bacterial strains

DNA encoding PduL20 was synthesized and cloned into the *BglII* and *Nde*I sites of pET14b (Sequence A1 (Appendix 3)). Genomic DNA used for PCRs was supplied by DSMZ (Table A1 (Appendix 2)). Primers (Table A2 (Appendix 2)), plasmids (Table A3 (Appendix 2)), and bacterial strains (Table A4 (Appendix 2)) used are available in the appendix. Molecular biology was carried out in *E. coli* JM109 strain, while all other experiments were carried out in *E. coli* BL21 Star (DE3) strain.

### 4.2 Growth of strains

BL21 Star (DE3) competent cells were transformed with appropriate plasmids. LB supplemented with ampicillin (100 mg/L) and chloramphenicol (34 mg/L) in baffled flasks was inoculated from an overnight starter culture. Cells were grown at 37°C with shaking to an OD600 ∼0.4, and protein production was induced by addition of IPTG to a final concentration of 400 μM. Cultures were incubated for 4 hr (confocal) or overnight at 19°C with shaking.

### 4.3 TEM analysis

Cells were harvested by centrifugation at 3,000 *g* for 10 min. The cell pellet was resuspended in 2 ml 2.5% (w/v) glutaraldehyde in 100 mM sodium cacodylate buffer, pH 7.2, (CAB) and fixed for 2 hr with gentle rotating (20 rpm). Cells were pelleted by centrifugation at 6,000 *g* for 2 min and were washed twice for 10 min with 100 mM CAB. Cells were postfixed with 1% (w/v) osmium tetroxide in 100 mM CAB for 2 hr and subsequently washed twice with ddH_2_O. Cells were dehydrated by incubation in an ethanol gradient, 50% EtOH for 10 min, 70% EtOH overnight, and 90% EtOH for 10 min followed by three 10‐min washes in 100% dry EtOH. Cells were then washed twice with propylene oxide for 15 min. Cell pellets were embedded by resuspension in 1 ml of a 1:1 mix of propylene oxide and Agar LV Resin and incubated for 30 min with rotation. Cell pellets were infiltrated twice in 100% Agar LV resin. The cell pellet was resuspended in fresh resin and transferred to a 1‐mL BEEM embedding capsule, centrifuged for 5 min at 3,000 *g* to concentrate the cells to the tip of the mold, and incubated for 20 hr at 60°C to polymerize.

Samples were ultrathin‐sectioned on an RMC MT‐XL ultramicrotome with a diamond knife (DiATOME 45°). Sections (60–70 nm) were collected on uncoated 300‐mesh copper grids. Grids were stained by incubation in 4.5% (w/v) uranyl acetate in 1% (v/v) acetic acid solution for 45 min followed by washing in a stream of ddH_2_O. Grids were then stained with Reynolds lead citrate for 7 min followed by washing in a stream of ddH_2_O.

Electron microscopy was performed using a JEOL‐1230 transmission electron microscope equipped with a Gatan multiscan digital camera operated at an accelerating voltage of 80 kV.

### 4.4 Purification of BMCs

Cells were harvested by centrifugation at 2,683 *g*. A 1 g wet cell pellet was resuspended in 20 ml Yeast Protein Extraction Reagent (Thermo Scientific) supplemented with Protease Inhibitor Cocktail Tablets, EDTA‐Free (Sigma‐Aldrich) and 500 Units Benzonase^®^ Nuclease (Merck) and incubated for 3 hr at room temperature with gentle shaking. Cell lysate was pelleted by centrifugation for 5 min at 11,300 *g*, and the pellet was resuspended in 2 ml of 20 mM Tris‐HCl, pH 8.0, 20 mM NaCl. The suspension was centrifuged at 4°C for 5 min at 11,000 *g*, and the supernatant was collected. The NaCl concentration was increased to 80 mM with 5 M NaCl, and this was then centrifuged at 4°C for 5 min at 11,000 *g*. The pellet was resuspended in 1 ml of 20 mM Tris‐HCl, pH 8.0, and was clarified by centrifugation at 4°C for 5 min at 11,000 *g*. The supernatant contains microcompartments was collected for analysis.

### 4.5 Pyruvate decarboxylase activity assay

Protein purification was carried out as described previously (Lee, Mantell, et al., 2018). Purified protein was buffer exchanged using a PD10 column (GE Healthcare) into 50 mM Na‐phosphate pH 7.0, 5 mM MgSO_4_, 0.1 mM thiamine pyrophosphate buffer. Enzyme concentration was estimated using absorbance at 280 nm and diluted to a stock concentration of 0.1 mg/ml. PDC activity was measured using an alcohol dehydrogenase coupled assay (Gounaris, Turkenkopf, Buckwald, & Young, 1971). Briefly, pyruvate is decarboxylated by PDC leading to the production of acetaldehyde which is subsequently reduced by alcohol dehydrogenase (ADH) using NADH. The oxidation of NADH is measured at 340 nm, and the rate of the reaction is calculated using the Michaelis–Menten equation. Reactions contained 0.15 mM NADH, 20U ADH (ADH from *S. cerevisiae*; Sigma), 1 µg PDC, 50 µM–10 mM Pyruvate, which was added last. All measurements were carried out at 25°C in 50 mM Na‐phosphate buffer, pH 7.0, containing 5 mM MgSO_4_ and 0.1 mM thiamine pyrophosphate.

### 4.6 Confocal imaging

Following growth and induction of protein expression, 1 ml of cells were harvested by centrifugation at 3,000 *g*. The resulting cell pellet was washed 3 times in PBS before incubation for 15 min in 2% (w/v) formaldehyde in PBS, and cells were then washed a further 3 times in PBS. Cells (10 μl) were pipetted onto a 1.5 thickness coverslip before being inverted onto a drop of ProLong Gold Antifade Mountant (Life Technologies) on a glass slide. Slides were incubated at room temperature in the dark for 24 hr to cure.

Images were acquired on a Zeiss LSM 880 with Airyscan system. Excitation light (514 nm for mCitrine or 561 nm for mCherry) was provided by an argon lamp (514 nm) or HeNe laser (561 nm). Images were acquired using a 100× 1.46 NA oil immersion objective lens.

### 4.7 Immuno‐TEM

Strains were cultured as described previously; cells were harvested by centrifugation for 10 min at 3,000 *g*. The cell pellet was resuspended in 2% formaldehyde and 0.5% glutaraldehyde in 100 mM sodium cacodylate, pH 7.2, and incubated for 2 hr with gentle rotating. Cells were pelleted by centrifugation at 6,000 *g* for 2 min and were washed twice for 10 min with 100 mM sodium cacodylate, pH 7.2. This was followed by dehydration of the samples in an ethanol gradient, 50% EtOH for 10 min, 70% EtOH for 10 min, and 90% EtOH for 10 min, followed by three 15‐min washes in 100% EtOH. Cell pellets were then resuspended in 2 ml LR white resin and incubated overnight with rotation at room temperature after which the resin was changed and incubated for a further 6 hr. Cell pellets were resuspended in fresh resin and transferred to 1‐mL gelatine capsules and centrifuged at 4,000 *g* to pellet the cells at the tip. Samples were polymerized at 60°C for 24 hr. Samples were ultrathin‐sectioned on an RMC MT‐XL ultramicrotome with a diamond knife (DiATOME 45°), and sections (60–70 nm thick) were collected on 300‐mesh gold grids.

Grids were equilibrated in one drop of TBST (20 mM Tris‐HCl buffer, pH 7.2, containing 500 mM NaCl, 0.05% Tween 20 and 0.1% BSA) before being transferred into a drop of 2% BSA in TBST and incubated at room temperature for 30 min. Grids were then immediately transferred into a 20 μl drop of relevant primary antibody (rabbit anti‐PduA (Parsons et al., 2010) or mouse anti‐GFP, for detecting Citrine; Sigma‐Aldrich) and incubated for 1 hr. Grids were washed in a fresh drop of TBST followed by washing for 10 s in a stream of TBST. Grids were equilibrated in a drop of relevant secondary antibody (goat anti‐rabbit IgG 10 nm or goat anti‐mouse IgG 10 nm gold (Agar Scientific)) and then incubated for 30 min in a fresh drop. Excess antibody was removed by washing in two drops of TBST before washing in a stream of ddH_2_O and dried.

Grids were stained for 15 min in 4.5% uranyl acetate in 1% acetic acid solution followed by 2 washes in dH_2_O. Grids were then stained with Reynolds lead citrate for 3 min followed by a wash in ddH_2_O. Electron microscopy was performed using a JEOL‐1230 transmission electron microscope equipped with a Gatan multiscan digital camera at an accelerating voltage of 80 kV.

### 4.8 Tomography

Sections (250 nm) were cut from the existing blocks as described above. Gold fiducials (15 nm, Aurion, TomoSol solution) were applied to both surfaces of the sections. The sections were imaged at 200 kV in a Tecnai 20 TEM (FEI, the Netherlands), and double‐tilt series images acquired between −67° to +69.5° (first axis) and −66° to +64.5° (second axis) with 1.5° (above 45°) and 2° increments (below 45°). The pixel size on the 4k by 4k FEI Eagle camera was 0.74 nm. The resulting tomograms were reconstructed and combined using IMOD software (Kremer, Mastronarde, & McIntosh, 1996). The isolated microcompartments were segmented manually using the AMIRA software suite, as shown in Video S2 at https://doi.org/10.5281/zenodo.3611413. Only structures fully covered by the tomogram section were analyzed. AMIRA software animations were further used for visualizing and analyzing the data.

## CONFLICT OF INTEREST

None declared.

## AUTHOR CONTRIBUTIONS

Rokas Juodeikis and Martin J. Warren conceived the study. Rokas Juodeikis and Matthew J. Lee performed formal analysis. Paul Verkade, Derek N. Woolfson, and Martin J. Warren was responsible for funding acquisition. Rokas Juodeikis, Matthew J. Lee, Matthias Mayer, and Judith Mantell performed the investigation. Rokas Juodeikis, Matthew J. Lee, Judith Mantell, and Ian R. Brown designed the methodology. Paul Verkade, Derek N. Woolfson, and Martin J. Warren involved in the project administration. Derek N. Woolfson, Michael B. Prentice, and Martin J. Warren collected the resources. Paul Verkade, Derek N. Woolfson, Stefanie Frank, and Martin J. Warren supervised the study. Rokas Juodeikis, Matthew J. Lee, Judith Mantell, and Ian R. Brown performed visualization experiments. Rokas Juodeikis, Matthew J. Lee, Judith Mantell, and Martin J. Warren wrote the manuscript and were involved in the original draft preparation. Rokas Juodeikis, Matthew J. Lee, Judith Mantell, and Martin J. Warren wrote, reviewed, and edited the manuscript.

## ETHICS STATEMENT

None required.

## Data Availability

The data supporting the findings of this study are available from the corresponding author upon reasonable request. Supplementary Videos are available in the Zenodo repository at https://doi.org/10.5281/zenodo.3611413. Video S1: electron microscopy tilt series and tomographic reconstructions of cells producing recombinant Pdu BMCs with and without targeting peptide or control (His‐) tagged fvMT; Video S2: recombinant Pdu BMCs traced using Amira software; and Video S3: recombinant Pdu BMCs containing L20‐fvMT traced using Amira software.

## APPENDIX 2

**Table A1:** Genomic DNA used in this study

Organism	DSMZ Number
*Gluconacetobacter diazotrophicus*	DSM‐5601
*Zymobacter palmae*	DSM‐10491
*Zymomonas mobilis*	DSM‐424

**Table A2:** Primers used in this study

Primer Name	Sequence
FWGdPDCNde	GTACATATGACCTATACCGTTGGACGCTATCTC
RVGdPDCSpeSac	GATACTAGTTCAGAGCTCGCCCGCGCGCGGCTGGCGGGCGTTG
FWZmPDCNde	GTACATATGAGTTATACTGTCGGTACCTATTTAGCGGAG
RVZmPDCSpeSac	GTTACTAGTCTAGAGCTCGAGGAGCTTGTTAACAGGCTTACGGCTG
FWZpPDCNde	GATCATATGTATACCGTTGGTATGTACTTGGCAGAAC
RVZpPDCSpeSac	GTTACTAGTTTAGAGCTCCGCTTGTGGTTTGCGAGAGTTGGTAGCTG
Fv.fMT.FW.2	GTACATATGGCGGGCACTGGCTGCAAGATCTGGGAAGAC
Fv.fMT.RV	CATACTAGTCACTTGCCGCAGCCGCAGCAGTC

**Table A3:** Plasmids used in this study

Plasmid name	Description	Source
pET14b	Overexpression vector containing N‐terminal hexahistidine tag, modified to include an *Spe*I site 5′ of *Bam*HI	Novagen
pET14b‐D18	Overexpression vector containing an N‐terminal D18 targeting tag followed by a short amino acid linker (AMGSS) then a hexahistidine tag	Lee et al. (2016)
pET14b‐L20	Overexpression vector containing an N‐terminal L20 targeting tag (first 20 amino acids of PduL from *Citrobacter freundii*) followed by a short amino acid linker (AMGSS) then a hexahistidine tag Synthesised DNA sequence shown in Sequence A1 (Appendix 3). *BglII/NdeI* ligated into *BglII/NdeI* site of pET14b vector	This study
pET14b‐P18	Overexpression vector containing an N‐terminal P18 targeting tag followed by a short amino acid linker (PMGSS) then a hexahistidine tag	Lee et al. (2016)
pLysS	Basal expression suppressor	Novagen
pLysS‐PduABJKNU	pLysS containing genes required for the formation of empty BMCs	Parsons et al. (2010)
pET3a‐pduD	pET3a vector containing *pduD* from C*itrobacter freundii* ligated into *NdeI/SpeI* site	Parsons et al. (2010)
pET3a‐pduL	pET3a vector containing *pduL* from C*itrobacter freundii* ligated into *NdeI/SpeI* site	This study
pET3a‐pduP	pET3a vector containing *pduP* from C*itrobacter freundii* ligated into *NdeI/SpeI* site	This study
pET3a‐mCherryPduABB’JKNU	pET3a vector containing genes required for the formation of empty BMCs tagged with mCherry fluorescent protein	Parsons et al. (2010)
pET14b.GdPDC	PCR product of GdPDC ligated into *NdeI/SpeI* sites of pET14b	This study
pET.D18‐GdPDC	*NdeI/SpeI* fragment from pET14b.GdPDC ligated into *NdeI/SpeI* site of pET14b‐D18	This study
pET.L20‐GdPDC	*NdeI/SpeI* fragment from pET14b.GdPDC ligated into *NdeI/SpeI* site of pET14b‐L20	This study
pET.P18‐GdPDC	*NdeI/SpeI* fragment from pET14b.GdPDC ligated into *NdeI/SpeI* site of pET14b‐P18	This study
pET14b.ZmPDC	PCR product of ZmPDC ligated into *NdeI/SpeI* sites of pET14b	This study
pET.D18‐ZmPDC	*NdeI/SpeI* fragment from pET14b.ZmPDC ligated into *NdeI/SpeI* site of pET14b‐D18	This study
pET.L20‐ZmPDC	*NdeI/SpeI* fragment from pET14b.ZmPDC ligated into *NdeI/SpeI* site of pET14b‐L20	This study
pET.P18‐ZmPDC	*NdeI/SpeI* fragment from pET14b.ZmPDC ligated into *NdeI/SpeI* site of pET14b‐P18	This study
pET14b.ZpPDC	PCR product of ZpPDC ligated into *NdeI/SpeI* sites of pET14b	This study
pET.D18‐ZpPDC	*NdeI/SpeI* fragment from pET14b.ZpPDC ligated into *NdeI/SpeI* site of pET14b‐D18	This study
pET.L20‐ZpPDC	*NdeI/SpeI* fragment from pET14b.ZpPDC ligated into *NdeI/SpeI* site of pET14b‐L20	This study
pET.P18‐ZpPDC	*NdeI/SpeI* fragment from pET14b.ZpPDC ligated into *NdeI/SpeI* site of pET14b‐P18	This study
pET_CC_Di_A_Citrine	Plasmid containing *citrine* gene in the *NdeI/SpeI* site	Lee, Mantell, et al. (2018)
pET14b.Citrine	*NdeI/SpeI* fragment from pET_CC_Di_A_Citrine ligated into *NdeI/SpeI* site of pET14b	This study
pET.D18‐Citrine	*NdeI/SpeI* fragment from pET14b.Citrine ligated into *NdeI/SpeI* site of pET14b‐D18	This study
pET.L20‐Citrine	*NdeI/SpeI* fragment from pET14b.Citrine ligated into *NdeI/SpeI* site of pET14b‐L20	This study
pET.P18‐Citrine	*NdeI/SpeI* fragment from pET14b.Citrine ligated into *NdeI/SpeI* site of pET14b‐P18	This study
pET_CC_Di_A_mCherry	Plasmid containing *mCherry* gene in the *NdeI/SpeI* site	Lee, Mantell, et al. (2018)
pET14b.mCheery	*NdeI/SpeI* fragment from pET_CC_Di_A_mCherry ligated into *NdeI/SpeI* site of pET14b	This study
pET.D18‐mCheery	*NdeI/SpeI* fragment from pET14b.mCheery ligated into *NdeI/SpeI* site of pET14b‐D18	This study
pET.L20‐mCheery	*NdeI/SpeI* fragment from pET14b.mCheery ligated into *NdeI/SpeI* site of pET14b‐L20	This study
pET.P18‐mCheery	*NdeI/SpeI* fragment from pET14b.mCheery ligated into *NdeI/SpeI* site of pET14b‐P18	This study
pET14b.fvMT	PCR of the coding sequence of fvMT ligated into *NdeI/SpeI* site of pET14b	This study
pET.D18‐fvMT	*NdeI/SpeI* fragment from pET14b.fvMT ligated into *NdeI/SpeI* site of pET14b‐D18	This study
pET.L20‐fvMT	*NdeI/SpeI* fragment from pET14b.fvMT ligated into *NdeI/SpeI* site of pET14b‐L20	This study
pET.P18‐fvMT	*NdeI/SpeI* fragment from pET14b.fvMT ligated into *NdeI/SpeI* site of pET14b‐P18	This study

**Table A4:** Strains used in this study

Strain	Genotype	Source
JM109	endA1, recA1, gyrA96, thi, hsdR17 (rk^−^, mk^+^), relA1, supE44, Δ(lac‐proAB), [F′, traD36, proAB, laqIqZΔM15]	Promega
BL21 Star (DE3)	F^−^ ompT hsdSB (rB^−^ mB^−^) gal dcm (DE3)	Novagen

## APPENDIX 3

**Sequence A1.** Synthetic sequence used to construct pET14b‐L20>L20HAGATCTCGATCCCGCGAAATTAATACGACTCACTATAGGGAGACCACAACGGTTTCCCTCTAGAAATAATTTTGTTTAACTTTAAGAAGGAGATATCATGGATAAACAGCAACTGGAGACAACGGTCACCAAAGTTCTGGATGAAATGCGTGAGCGCGCCATGGGCAGCAGCCATCATCATCATCATCACAGCAGCGGCCTGGTGCCGCGCGGCAGCCATATG

## References

[mbo31010-bib-0001] Aussignargues, C. , Paasch, B. C. , Gonzalez‐Esquer, R. , Erbilgin, O. , & Kerfeld, C. A. (2015). Bacterial microcompartment assembly: The key role of encapsulation peptides. Communicative & Integrative Biology, 8, e1039755 10.1080/19420889.2015.1039755 26478774 PMC4594438

[mbo31010-bib-0002] Axen, S. D. , Erbilgin, O. , & Kerfeld, C. A. (2014). A Taxonomy of bacterial microcompartment loci constructed by a novel scoring method. PLoS Computational Biology, 10, e1003898 10.1371/journal.pcbi.1003898 25340524 PMC4207490

[mbo31010-bib-0003] Bobik, T. A. (2006). Polyhedral organelles compartmenting bacterial metabolic processes. Applied Microbiology and Biotechnology, 70, 517–525. 10.1007/s00253-005-0295-0 16525780

[mbo31010-bib-0004] Bobik, T. A. , Havemann, G. D. , Busch, R. J. , Williams, D. S. , & Aldrich, H. C. (1999). The propanediol utilization (*pdu*) operon of *Salmonella enterica* serovar Typhimurium LT2 includes genes necessary for formation of polyhedral organelles involved in coenzyme B_12_‐dependent 1, 2‐propanediol degradation. Journal of Bacteriology, 181, 5967–5975. 10498708 10.1128/jb.181.19.5967-5975.1999 PMC103623

[mbo31010-bib-0005] Bobik, T. A. , Lehman, B. P. , & Yeates, T. O. (2015). Bacterial microcompartments: Widespread prokaryotic organelles for isolation and optimization of metabolic pathways. Molecular Microbiology, 98, 193–207. 10.1111/mmi.13117 26148529 PMC4718714

[mbo31010-bib-0006] Brinsmade, S. R. , Paldon, T. , & Escalante‐Semerena, J. C. (2005). Minimal functions and physiological conditions required for growth of *Salmonella enterica* on ethanolamine in the absence of the metabolosome. Journal of Bacteriology, 187, 8039–8046. 10.1128/JB.187.23.8039-8046.2005 16291677 PMC1291257

[mbo31010-bib-0007] Cai, F. , Dou, Z. , Bernstein, S. L. , Leverenz, R. , Williams, E. B. , Heinhorst, S. , … Kerfeld, C. A. (2015) Advances in understanding carboxysome assembly in Prochlorococcus and Synechococcus implicate CsoS2 as a critical component. Life (Basel), 5, 1141–1171. 10.3390/life5021141 25826651 PMC4499774

[mbo31010-bib-0008] Cameron, J. C. , Wilson, S. C. , Bernstein, S. L. , & Kerfeld, C. A. (2013). Biogenesis of a bacterial organelle: The carboxysome assembly pathway. Cell, 155, 1131–1140. 10.1016/j.cell.2013.10.044 24267892

[mbo31010-bib-0009] Chen, A. H. , Robinson‐Mosher, A. , Savage, D. F. , Silver, P. A. , & Polka, J. K. (2013). The bacterial carbon‐fixing organelle is formed by shell envelopment of preassembled cargo. PLoS ONE, 8, e76127 10.1371/journal.pone.0076127 24023971 PMC3762834

[mbo31010-bib-0010] Cheng, S. , Liu, Y. , Crowley, C. S. , Yeates, T. O. , & Bobik, T. A. (2008). Bacterial microcompartments: Their properties and paradoxes. BioEssays, 30, 1084–1095. 10.1002/bies.20830 18937343 PMC3272490

[mbo31010-bib-0011] Fan, C. , & Bobik, T. A. (2011). The N‐terminal region of the medium subunit (PduD) packages adenosylcobalamin‐dependent diol dehydratase (PduCDE) into the Pdu microcompartment. Journal of Bacteriology, 193, 5623–5628. 10.1128/JB.05661-11 21821773 PMC3187188

[mbo31010-bib-0012] Fan, C. , Cheng, S. , Liu, Y. , Escobar, C. M. , Crowley, C. S. , Jefferson, R. E. , … Bobik, T. A. (2010). Short N‐terminal sequences package proteins into bacterial microcompartments. Proceedings of the National Academy of Sciences, 107, 7509–7514. 10.1073/pnas.0913199107 PMC2867708 20308536

[mbo31010-bib-0013] Fan, C. , Cheng, S. , Sinha, S. , & Bobik, T. A. (2012). Interactions between the termini of lumen enzymes and shell proteins mediate enzyme encapsulation into bacterial microcompartments. Proceedings of the National Academy of Sciences, 109, 14995–15000. 10.1073/pnas.1207516109 PMC3443165 22927404

[mbo31010-bib-0014] Frank, S. , Lawrence, A. D. , Prentice, M. B. , & Warren, M. J. (2013). Bacterial microcompartments moving into a synthetic biological world. Journal of Biotechnology, 163, 273–279. 10.1016/j.jbiotec.2012.09.002 22982517

[mbo31010-bib-0015] Gounaris, A. D. , Turkenkopf, I. , Buckwald, S. , & Young, A. (1971). Pyruvate decarboxylase. I. Protein dissociation into subunits under conditions in which thiamine pyrophosphate is released. Journal of Biological Chemistry, 246, 1302–1309. 5545074

[mbo31010-bib-0016] Iancu, C. V. , Ding, H. J. , Morris, D. M. , Dias, D. P. , Gonzales, A. D. , Martino, A. , & Jensen, G. J. (2007). The structure of isolated *Synechococcus* strain WH8102 carboxysomes as revealed by electron cryotomography. Journal of Molecular Biology, 372, 764–773. 10.1016/j.jmb.2007.06.059 17669419 PMC2453779

[mbo31010-bib-0017] Jakobson, C. M. , Tullman‐Ercek, D. , Slininger, M. F. , & Mangan, N. M. (2017). A systems‐level model reveals that 1,2‐Propanediol utilization microcompartments enhance pathway flux through intermediate sequestration. PLoS Computational Biology, 13, e1005525. 10.1371/journal.pcbi.1005525 PMC5438192 28475631

[mbo31010-bib-0018] Kagi, J. H. , & Schaffer, A. (1988). Biochemistry of metallothionein. Biochemistry, 27, 8509–8515. 10.1021/bi00423a001 3064814

[mbo31010-bib-0019] Kerfeld, C. A. , Aussignargues, C. , Zarzycki, J. , Cai, F. , & Sutter, M. (2018). Bacterial microcompartments. Nature Reviews Microbiology, 16, 277–290. 10.1038/nrmicro.2018.10 29503457 PMC6022854

[mbo31010-bib-0020] Kerfeld, C. A. , Heinhorst, S. , & Cannon, G. C. (2010). Bacterial microcompartments. Annual Reviews of Microbiology, 64, 391–408. 10.1146/annurev.micro.112408.134211 20825353

[mbo31010-bib-0021] Kerfeld, C. A. , & Melnicki, M. R. (2016). Assembly, function and evolution of cyanobacterial carboxysomes. Current Opinions in Plant Biology, 31, 66–75. 10.1016/j.pbi.2016.03.009 27060669

[mbo31010-bib-0022] Kerfeld, C. A. , Sawaya, M. R. , Tanaka, S. , Nguyen, C. V. , Phillips, M. , Beeby, M. , & Yeates, T. O. (2005). Protein structures forming the shell of primitive bacterial organelles. Science, 309, 936–938. 10.1126/science.1113397 16081736

[mbo31010-bib-0023] Kim, E. Y. , & Tullman‐Ercek, D. (2013). Engineering nanoscale protein compartments for synthetic organelles. Current Opinion in Biotechnology, 24, 627–632. 10.1016/j.copbio.2012.11.012 23273660

[mbo31010-bib-0024] Kim, E. Y. , & Tullman‐Ercek, D. (2014). A rapid flow cytometry assay for the relative quantification of protein encapsulation into bacterial microcompartments. Biotechnology Journal, 9, 348–354. 10.1002/biot.201300391 24323373

[mbo31010-bib-0025] Kinney, J. N. , Salmeen, A. , Cai, F. , & Kerfeld, C. A. (2012). Elucidating essential role of conserved carboxysomal protein CcmN reveals common feature of bacterial microcompartment assembly. Journal of Biological Chemistry, 287, 17729–17736. 10.1074/jbc.M112.355305 22461622 PMC3366800

[mbo31010-bib-0026] Kremer, J. R. , Mastronarde, D. N. , & McIntosh, J. R. (1996). Computer visualization of three‐dimensional image data using IMOD. Journal of Structural Biology, 116, 71–76. 10.1006/jsbi.1996.0013 8742726

[mbo31010-bib-0027] Lawrence, A. D. , Frank, S. , Newnham, S. , Lee, M. J. , Brown, I. R. , Xue, W.‐F. , … Warren, M. J. (2014). Solution structure of a bacterial microcompartment targeting peptide and its application in the construction of an ethanol bioreactor. ACS Synthetic Biology, 3, 454–465. 10.1021/sb4001118 24933391 PMC4880047

[mbo31010-bib-0028] Lee, M. J. , Brown, I. R. , Juodeikis, R. , Frank, S. , & Warren, M. J. (2016). Employing bacterial microcompartment technology to engineer a shell‐free enzyme‐aggregate for enhanced 1,2‐propanediol production in *Escherichia coli* . Metabolic Engineering, 36, 48–56. 10.1016/j.ymben.2016.02.007 26969252 PMC4909751

[mbo31010-bib-0029] Lee, M. J. , Mantell, J. , Hodgson, L. , Alibhai, D. , Fletcher, J. M. , Brown, I. R. , … Warren, M. J. (2018). Engineered synthetic scaffolds for organizing proteins within the bacterial cytoplasm. Nature Chemical Biology, 14, 142–147. 10.1038/nchembio.2535 29227472

[mbo31010-bib-0030] Lee, M. J. , Palmer, D. J. , & Warren, M. J. (2018). Biotechnological advances in bacterial microcompartment technology. Trends in Biotechnology, 37, 325–336. 10.1016/j.tibtech.2018.08.006 30236905

[mbo31010-bib-0031] Liang, M. , Frank, S. , Lunsdorf, H. , Warren, M. J. , & Prentice, M. B. (2017). Bacterial microcompartment‐directed polyphosphate kinase promotes stable polyphosphate accumulation in *E. coli* . Biotechnol Journal, 12, 1600415. 10.1002/biot.201600415 28105684

[mbo31010-bib-0032] Liu, Y. , Jorda, J. , Yeates, T. O. , & Bobik, T. A. (2015). The PduL phosphotransacylase is used to recycle coenzyme A within the Pdu microcompartment. Journal of Bacteriology, 14, 23922–23928. 10.1128/JB.00056-15 PMC4524188 25962918

[mbo31010-bib-0033] Mayer, M. J. , Juodeikis, R. , Brown, I. R. , Frank, S. , Palmer, D. J. , Deery, E. , … Warren, M. J. (2016). Effect of bio‐engineering on size, shape, composition and rigidity of bacterial microcompartments. Scientific Reports, 6, 36899 10.1038/srep36899 27845382 PMC5109269

[mbo31010-bib-0034] Mohajerani, F. , & Hagan, M. F. (2018). The role of the encapsulated cargo in microcompartment assembly. PLoS Computational Biology, 14, e1006351 10.1371/journal.pcbi.1006351 30063715 PMC6086489

[mbo31010-bib-0035] Morris, C. A. , Nicolaus, B. , Sampson, V. , Harwood, J. L. , & Kille, P. (1999). Identification and characterization of a recombinant metallothionein protein from a marine alga, *Fucus vesiculosus* . Biochemical Journal, 338, 553–560. 10.1042/bj3380553 10024535 PMC1220085

[mbo31010-bib-0036] Parsons, J. B. , Frank, S. , Bhella, D. , Liang, M. , Prentice, M. B. , Mulvihill, D. P. , & Warren, M. J. (2010). Synthesis of empty bacterial microcompartments, directed organelle protein incorporation, and evidence of filament‐associated organelle movement. Molecular Cell, 38, 305–315. 10.1016/j.molcel.2010.04.008 20417607

[mbo31010-bib-0037] Penrod, J. T. , & Roth, J. R. (2006). Conserving a volatile metabolite: A role for carboxysome‐like organelles in Salmonella enterica. Journal of Bacteriology, 188, 2865–2874. 10.1128/JB.188.8.2865-2874.2006 16585748 PMC1447003

[mbo31010-bib-0038] Perlmutter, J. D. , Mohajerani, F. , & Hagan, M. F. (2016) Many‐molecule encapsulation by an icosahedral shell. eLife, 5, e14078 10.7554/eLife.14078.001 27166515 PMC4947392

[mbo31010-bib-0039] Rae, B. D. , Long, B. M. , Badger, M. R. , & Price, G. D. (2013). Functions, compositions, and evolution of the two types of carboxysomes: Polyhedral microcompartments that facilitate CO2 fixation in cyanobacteria and some proteobacteria. Microbiology and Molecular Biology Reviews: MMBR, 77, 357–379. 10.1128/MMBR.00061-12 24006469 PMC3811607

[mbo31010-bib-0040] Sampson, E. M. , & Bobik, T. A. (2008). Microcompartments for B_12_‐dependent 1,2‐propanediol degradation provide protection from DNA and cellular damage by a reactive metabolic intermediate. Journal of Bacteriology, 190, 2966–2971. 10.1128/JB.01925-07 18296526 PMC2293232

[mbo31010-bib-0041] Sutter, M. , Greber, B. , Aussignargues, C. , & Kerfeld, C. A. (2017). Assembly principles and structure of a 6.5‐MDa bacterial microcompartment shell. Science, 356, 1293–1297. 10.1126/science.aan3289 28642439 PMC5873307

[mbo31010-bib-0042] Tanaka, S. , Kerfeld, C. A. , Sawaya, M. R. , Cai, F. , Heinhorst, S. , Cannon, G. C. , & Yeates, T. O. (2008). Atomic‐level models of the bacterial carboxysome shell. Science, 319, 1083–1086. 10.1126/science.1151458 18292340

[mbo31010-bib-0043] Weber, B. , Greenan, G. , Prohaska, S. , Baum, D. , Hege, H. C. , Muller‐Reichert, T. , … Verbavatz, J. M. (2012). Automated tracing of microtubules in electron tomograms of plastic embedded samples of *Caenorhabditis elegans* embryos. Journal of Structural Biology, 178, 129–138. 10.1016/j.jsb.2011.12.004 22182731

[mbo31010-bib-0044] Yeates, T. O. , Kerfeld, C. A. , Heinhorst, S. , Cannon, G. C. , & Shively, J. M. (2008). Protein‐based organelles in bacteria: Carboxysomes and related microcompartments. Nature Reviews Microbiology, 6, 681–691. 10.1038/nrmicro1913 18679172

